# Oncological risk of proximal gastrectomy for proximal advanced gastric cancer after neoadjuvant chemotherapy

**DOI:** 10.1186/s12885-024-11993-5

**Published:** 2024-02-23

**Authors:** Yonghe Chen, Xiaojiang Chen, Yi Lin, Shenyan Zhang, Zhiwei Zhou, Junsheng Peng

**Affiliations:** 1 Department of General Surgery, The Sixth Affiliated Hospital, Sun Yat-sen University, 26 Yuancun Erheng Road, 510655 Guangzhou, China; 2 Guangdong Provincial Key Laboratory of Colorectal and Pelvic Floor Diseases, The Sixth Affiliated Hospital, Sun Yat-sen University, 510655 Guangzhou, Chinaf China; 3 Biomedical Innovation Center, The Sixth Affiliated Hospital, Sun Yat-sen University, 510655 Guangzhou, China; 4 Department of Gastric Surgery, Sun Yat-Sen University Cancer Center, 510060 Guangzhou, China; 5 Department of Pathology, The Sixth Affiliated Hospital, Sun Yat-sen University, 510655 Guangzhou, China

**Keywords:** Proximal gastric cancer, Proximal gastrectomy, Neoadjuvant chemotherapy, Tumor regression grade

## Abstract

**Purpose:**

This study assesses the metastasis rate of the key distal lymph nodes (KDLN) that are not routinely dissected in proximal gastrectomy, aiming to explore the oncological safety of proximal gastrectomy for upper gastric cancer who underwent neoadjuvant chemotherapy.

**Methods:**

We analyzed a cohort of 150 patients with proximal locally advanced gastric cancer (cT3/4 before chemotherapy) from two high-volume cancer centers in China who received preoperative neoadjuvant chemotherapy (NAC) and total gastrectomy with lymph node dissection. Metastasis rate of the KDLN (No.5/6/12a) and the risk factors were analyzed.

**Results:**

Key distal lymph node metastasis was detected in 10% (15/150) of patients, with a metastasis rate of 6% (9/150) in No. 5 lymph nodes, 6.7% (10/150) in No. 6 lymph nodes, and 2.7% (2/75) in No. 12a lymph nodes. The therapeutic value index of KDLN as one entity is 5.8. Tumor length showed no correlation with KDLN metastasis, while tumor regression grade (TRG) emerged as an independent risk factor (OR: 1.47; p-value: 0.04). Of those with TRG3 (no response to NAC), 80% (12/15) was found with KDLN metastasis.

**Conclusion:**

For cT3/4 proximal locally advanced gastric cancer patients, the risk of KDLN metastasis remains notably high even after NAC. Therefore, proximal gastrectomy is not recommended; instead, total gastrectomy with thorough distal lymphadenectomy is the preferred surgical approach.

## Introduction

Gastric cancer is the fifth most common malignancy and the fourth leading cause of cancer-related death worldwide [1]. In recent years, the prevalence of upper gastric cancer is rising rapidly. Total gastrectomy with D2 lymphadenectomy is the standard surgical procedure [2]. However, total gastrectomy leads to decreased food intake and reduced body weight, impairing not only the quality of life but also survival [3]. Proximal gastrectomy can improve postoperative nutritional intake by preserving the distal part of the stomach [4]. Currently, it is only indicated for early gastric cancer where lymph node metastasis is rare. For the advanced cases, there is the concern of potential cancer remaining in the distal lymph nodes, especially the No. 4d/5/6/12a lymph nodes (referred to as the key distal lymph nodes, KDLN), which may eventually cause early tumor recurrence, hindering survival. Hence, it remains uncertain whether it’s oncologically safe to perform proximal gastrectomy for these patients [5]. In a previous study, Masahiro et al. found that the possibility of KDLN metastasis is extremely low, even in T2/3 cases [6], giving the conclusion that proximal gastrectomy is an oncologically safe choice for specific advanced cases with tumors in the upper part. However, in their study, the patients that received neoadjuvant chemotherapy were excluded. Nowadays, neoadjuvant chemotherapy has become a popular treatment strategy, especially for tumors located in the esophagogastric junction and the upper part of the stomach [7–9]. NAC could potentially downstage the tumors and eliminate micro metastasis sites in the lymph nodes, giving rise to one question: Is it safe to perform proximal gastrectomy for proximal advanced gastric cancer after NAC?

To answer this question, we reviewed the patients with tumors located in the upper part of the stomach, who eventually received NAC and total gastrectomy from 2 high-volume cancer centers. The metastasis status of the perigastric lymph nodes, especially the KDLN, was evaluated, aiming to explore the oncological safety of performing proximal gastrectomy for advanced gastric cancer patients after NAC.

## Methods

### Study design, inclusion, and exclusion criteria

All clinical data were extracted from the gastric cancer databases of two institutions: The Sixth Affiliated Hospital, Sun Yat-sen University (Guangzhou, China), and Sun Yat-Sen University Cancer Center (Guangzhou, China). This study was conducted with the approval of the Ethics Committee Board of our institution (Approval number: 2022ZSLYEC-020).

#### Inclusion criteria

(i) Age between 18 and 80 years, of any sex; (ii) Histological diagnosis of gastric/esophagogastric junction adenocarcinoma in the upper part of the stomach; (iii) Received neoadjuvant chemotherapy; (iv) Received total gastrectomy with standardized D2 lymphadenectomy.

#### Exclusion criteria

(i) Insufficient information regarding perigastric grouped station lymph node metastasis status; (ii) Diagnosis of remnant gastric cancer; (iii) Presence of concurrent malignant tumors; (iv) Received concurrent preoperative radiotherapy.

These criteria were applied to select the study participants and ensure data quality and relevance.

### Pre-intervention staging, NAC regimen, and surgery

Clinical T and N stages were determined using thoracic-abdominal-pelvic computed tomography, in accordance with the American Joint Committee on Cancer staging criteria [10–14].

- Clinical T1 indicates invasion of the mucosa or submucosa.
- Clinical T2 indicates invasion of the muscularis propria.
- Clinical T3 indicates invasion of the subserosal connective tissue without invading the visceral peritoneum.
- Clinical T4 indicates invasion of the serosa (visceral peritoneum) with or without involvement of adjacent structures/organs.

The clinical N stage classified as N0-N3 based on the number of metastatic lymph nodes. The diameter of circular enlarged lymph node > 1 cm in computed tomography was identified as suspicious metastatic lymph node.

The preoperative neoadjuvant chemotherapy (NAC) regimen consisted of a combination of docetaxel (or Paclitaxel), platinum (oxaliplatin or cisplatin), and fluorouracil (or its analogue such as capecitabine). None of the patients received immunotherapy or preoperative radiotherapy during the preoperative treatment. The dose of NAC for each patient was determined by the treating oncologists in accordance with the National Comprehensive Cancer Network guidelines. Following the completion of NAC, all patients underwent standard total gastrectomy with D2 lymph node dissection, adhering to the guidelines outlined by the Japanese Gastric Cancer Association (JGCA). The D2 lymph node dissection for total gastrectomy involved the clearance of lymph nodes including No.1–7, 8a, 9, 11p, 11d, and 12a lymph nodes [2].

### Specimen assessment

Lymph nodes were retrieved from the gross specimens manually. Suspicious lymph nodes were initially identified through visual inspection and palpation and subsequently confirmed under microscopic examination. Lymph node labeling was based on their anatomical locations and their relationship to the perigastric vessels. At Sun Yat-Sen University Cancer Center, this procedure was performed by the surgeons on the fresh specimens instantly after surgical resection. At the Sixth Affiliated Hospital of Sun Yat-Sen University, this procedure was performed by the pathologists on the formalin-fixed specimens. Pathological staging was determined according to the AJCC TNM staging system [14].

### Therapeutic value index of lymph node dissection

The therapeutic value index presented by Sasako et al [15] was used to evaluate the therapeutic value of dissection at each specific LN station. The calculating formula is: Therapeutic value index of specific lymph node station **=** metastasis rate **×** 3-year overall survival rate in patients with metastasis.

### Histopathologic response and post-surgery complication evaluation

The histological response to NAC was assessed using the Tumor Regression Grade (TRG), which is based on the presence of viable tumor cells within the tumor [16, 17]. The TRG grading system is as follows:

- Grade 0 (Pathological complete response): No tumor cells remained.
- Grade 1 (Major response): Scattered single tumor cells remained.
- Grade 2 (Minor response): Clustered tumor cells remained with fibrosis.
- Grade 3 (No response): Extensive tumor cells remained.

### Follow-up

All patients were recommended with regular follow-up assessments during the first 2 years, with appointments every 3 months followed by subsequent appointments every 6 months. Each follow-up examination consisted of a thorough review of medical history, physical examination, routine blood tests, comprehensive biochemical analyses, and CT scans. The hospital’s follow-up office conducted these assessments through telephone calls or mail correspondence to gather information on the patients’ health status and survival. Overall survival (OS) was defined as the duration from the day of surgery until either the date of death or the final follow-up date.

### Data analysis

The normality of the data was assessed using the Kolmogorov-Smirnov test and normal probability plots. Parameters that did not follow a normal distribution were presented as the median (interquartile range) and analyzed using non-parametric tests, such as the Mann-Whitney test or Kruskal-Wallis test, as appropriate. Parameters that exhibited a normal distribution were presented as mean ± standard deviation and analyzed using Student’s t-test. In the multivariate analysis, parameters with statistical significance and clinical relevance were selected into the logistic regression model, those with a P-value less than 0.05 were consider independent factor. All statistical analyses were conducted utilizing SPSS software version 25.0 (IBM, Armonk, NY, USA) and R software version 4.1.1 (The R Foundation for Statistical Computing, Vienna, Austria; www.r-project.org).

## Results

### Patient characteristics

As presented in Table 1, the study included a total of 150 eligible patients with locally advanced gastric cancer (LAGC). Among them, **10% (15/150) were ultimately diagnosed with metastasis in key distal lymph nodes**, as confirmed by pathological evidence following total gastrectomy. The patient cohort was predominantly composed of elderly males, with a median age of 61 years. All patients had adenocarcinoma located in the upper part of the stomach, with advanced clinical T3/4 stages, predominantly poorly differentiated adenocarcinoma. Following a median of 3 cycles of neoadjuvant chemotherapy, the median longitudinal tumor diameter decreased from 5.75 cm to 3.50 cm. Subsequently, all patients underwent total gastrectomy with standardized lymphadenectomy.

**Table 1:** Patients’ characteristics, risk factors for key distal lymph node metastasis in univariate analysis and multivariate analysis

Characteristics		Total (*N* = 150)	Key distal lymph nodes negative (*N* = 135)	Key distal lymph nodes positive (*N* = 15)	p-value (Univariate analysis)	Odd ratio and p-value (Multivariate logistic regression analysis)
Sex (%)	Male	122 (81.3)	109 (80.7)	13 (86.7)	0.834	NA
	Female	28 (18.7)	26 (19.3)	2 (13.3)		
Age		61 [55.25,67]	61 [55,67]	61 [57,65]	0.728	NA
Tumor differentiation (%)	Well	7 (4.7)	7 (5.2)	0 (0.0)	0.02	Odd Ratio: 18.44; p-value: 0.99
	Moderately	41 (27.3)	41 (30.4)	0 (0.0)		
	Poorly	102 (68.0)	87 (64.4)	15 (100.0)		
Tumor longitudinal diameter before chemotherapy		5.75 [4.62,8]	6 [5, 8]	5 [3, 8]	0.269	NA
Clinical T stage	T3	62(41.3)	61(45.2)	1(6.7)	0.009	Odd Ratio: 1.79; p-value: 0.14
	T4	88(58.7)	74(54.8)	14(93.3)		
Clinical N stage	N0	10(6.7)	9(6.7)	1(6.7)	0.188	
	N1	59(39.3)	54(40.0)	5(33.3)		
	N2	71(47.3)	65(48.1)	6(40.0)		
	N3	10(6.7)	7(5.2)	3(20.0)		
Neoadjuvant chemotherapy regimens	SOX	35(23.3)	32(23.7)	3(20.0)	0.09	NA
	CAPOX	48(32.0)	45(33.3)	3(20.0)		
	Folfox	6(4.0)	5(3.7)	1(6.7)		
	mFLOT	43(28.7)	40(29.6)	3(20.0)		
	Others	18(12.0)	13(9.6)	5(33.3)		
Cycles received before resection surgery		3 [3, 4]	3 [3, 4]	2 [2,3.50]	0.022	Odd Ratio: -0.06; p-value: 0.85
Tumor longitudinal diameter after chemotherapy		3.50 [2.50,5]	3.50 [2.50,5]	4 [3, 7]	0.117	NA
R0 resection		143 (95.3)	128 (94.8)	15 (100.0)	0.796	
Tumor regression grade* (%)	Grade 0	19 (12.7)	19 (14.1)	0 (0.0)	< 0.001	Odd Ratio: 1.47; p-value: 0.04
	Grade 1	22 (14.7)	21 (15.6)	1 (6.7)		
	Grade 2	81 (54.0)	79 (58.5)	2 (13.3)		
	Grade 3	28 (18.7)	16 (11.9)	12 (80.0)		
ypT stage (%)	ypT0	19 (12.7)	18 (13.3)	0 (0.0)	0.033	Odd Ratio: 0.20; p-value: 0.72
	ypT1	11 (7.3)	11 (8.1)	0 (0.0)		
	ypT2	13 (8.7)	14 (10.4)	0 (0.0)		
	ypT3	89 (59.3)	79 (58.5)	10 (66.7)		
	ypT4	18 (12.0)	12 (8.9)	5 (33.3)		
ypN stage (%)	ypN0	80(53.3)	80(59.3)	0(0.0)	< 0.001	Odd Ratio: 0.93; p-value: 0.03
	ypN1	27(18.0)	25(18.5)	2(13.3)		
	ypN2	18(12.0)	14(10.4)	4(26.7)		
	ypN3	25(16.7)	16(11.9)	9(60.0)		
Number of total lymph node examined		37 [26.25,48.75]	37 [26,48]	44 [29.50,55]	0.163	NA
Adjuvant chemotherapy	SOX	32(21.3)	27(20.0)	5(33.3)	0.873	NA
	CAPOX	48(32.0)	45(33.3)	3(20.0)		
	mFLOT	12(8.0)	12(8.9)	0(0.0)		
	Folfox	11(7.3)	10(7.4)	1(6.7)		
	Oral S-1	11(7.3)	10(7.4)	1(6.7)		
	Other	36(24.0)	31(23.0)	5(33.3)		
Adjuvant cycles		3 [1, 4]	3 [1, 4]	2 [1, 3]	0.179	
Number of total lymph node examined		37 [26.25,48.75]	37 [26,48]	44 [29.50,55]	0.163	NA

**Tumor regression grade**: Grade 0 (pathological complete response); Grade 1 (major response); Grade 2 (minor response); Grade 3 (no response or progression)

**OR**: odd ratio

### Perigastric lymph node metastasis rate, therapeutic index, and risk factors

**Fig. 1:**
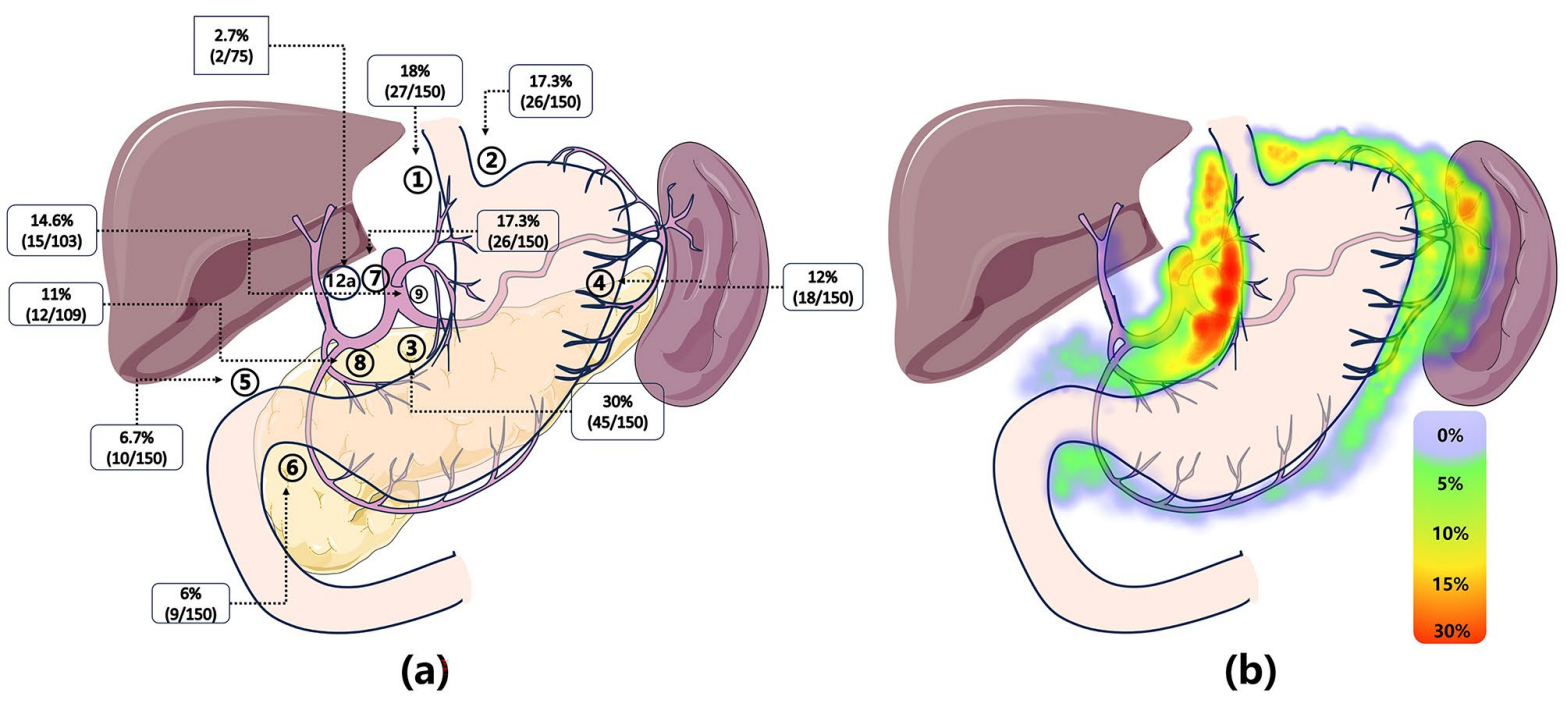
Q1 (**a**) This data map illustrates the metastasis rate of perigastric lymph nodes in proximal gastric cancer patients after neoadjuvant chemotherapy. Lymph nodes surrounding the proximal part of the stomach, such as No. 1/2/3/7, exhibit the highest metastasis rate (17.3%~30%). Key distal lymph nodes, including No. 5/6/12a, have a collective metastasis rate of 10%. (**b**) This heatmap provides a visualization of the metastasis rate of grouped perigastric lymph nodes

As presented in Table 2, all resected specimens underwent thorough dissection and examination to assess perigastric grouped station lymph nodes. Pathologically positive lymph nodes were confirmed in almost half of the patients (46.7%, 70/150). Figure 1 illustrates that lymph nodes surrounding the proximal part of the stomach, such as No. 1/2/3/7, exhibited the highest metastasis rates. The No.10 lymph nodes exhibited a metastasis rate as high as 20.8%. However, it’s important to note that this data may be biased. Only 24 patients had their splenic hilar lymph nodes (No.10 lymph nodes) dissected, all of these patients had their No.10 lymph nodes dissected due to high suspect of metastasis by CT scans.

**Table 2:** Metastasis rate of grouped lymph node stations by pathological examination

Lymph node station	Metastasis rate (%)			3 years survival rate with nodal metastasis patients (%)			Therapeutic value index (Metastasis rate×3 years survival rate with nodal metastasis patients×100)		
	Tumor length > = 4CM	Tumor length < 4CM	Total	Tumor length > = 4CM	Tumor length < 4CM	Total	Tumor length > = 4CM	Tumor length < 4CM	Total
**KDLN**	**7.2% (10/139)**	**45.5% (5/11)**	**10% (15/150)**	**76.2%**	**53.3%**	**58.4%**	**5.5**	**24.3**	**5.8**
1	15.8% (22/139)	45.5% (5/11)	18% (27/150)	45.7%	NA	42.1%	7.2	NA	7.6
2	15.8% (22/139)	36.4% (4/11)	17.3% (26/150)	46.3%	NA	39%	7.3	NA	6.7
3	28.1% (39/139)	54.5% (6/11)	30% (45/150)	65.4%	NA	59.5%	18.4	NA	17.9
4	10.8% (15/139)	27.3% (3/11)	12% (18/150)	46.4%	NA	35.3%	5	NA	4.2
5	4.3% (6/139)	27.3% (3/11)	6% (9/150)	66.7%	66.7%	66.7%	2.9	18.2	4
6	4.3% (6/139)	36.4% (4/11)	6.7% (10/150)	83.3%	NA	53.3%	3.6	NA	3.6
7	17.3% (24/139)	18.2% (2/11)	17.3% (26/150)	50.5%	NA	46.4%	8.7	NA	8
8	9.2% (9/98)	27.3% (3/11)	11% (12/109)	41.7%	NA	32.1%	3.8	NA	3.5
9	14% (13/93)	20% (2/10)	14.6% (15/103)	54.9%	NA	63.5%	7.7	NA	9.3
10	20% (4/20)	25% (1/4)	20.8% (5/24)	NA	NA	NA	NA	NA	NA
11	5.2% (4/77)	22.2% (2/9)	7% (6/86)	NA	NA	NA	NA	NA	NA
12a	3% (2/67)	0% (0/8)	2.7% (2/75)	NA	NA	NA	NA	NA	NA
All	45.3% (63/139)	63.6% (7/11)	46.7% (70/150)	76.0%	56.3%	73.0%	34.4	35.8	34.1

**KDLN**: Either of No.5/6/12a lymph node is found with metastasis tumor cells

**Key distal lymph node metastasis was observed in 10% of patients (15/150)**. Among these cases, 6% had No.5 lymph node metastasis, 6.7% had No.6 lymph node metastasis, and 2.7% had No.12 lymph node metastasis. Additionally, 12% had No.4 lymph node metastasis; however, detailed information regarding No.4a/4sb/4d was unavailable.

As for the therapeutic index, the No. 3/9/7 exhibit the highest therapeutic value, with index ranging from 8 to 17.3. Therapeutic value of the No.5/6/12a lymph was relatively low, ranging from 0 to 3.6. However, when merged into one entity (KDLN, considered positive when either of the No.5/6/12a is found positive), the therapeutic value rise up to 5.8.

Several risk factors for key distal lymph node metastasis were identified, including poor tumor differentiation, inadequate neoadjuvant chemotherapy administration, unsatisfactory tumor regression grade (TRG), and more advanced pathological ypT/N stage. In the multivariate analysis, unsatisfactory TRG and advance ypTN stages emerged as significant risk factors, especially TRG. 80% of patients who exhibited no response to neoadjuvant therapy (TRG3) were found with KDLN metastasis. Interestingly, the tumor length did not seem to be correlated with KDLN metastasis.

**Fig. 2:**
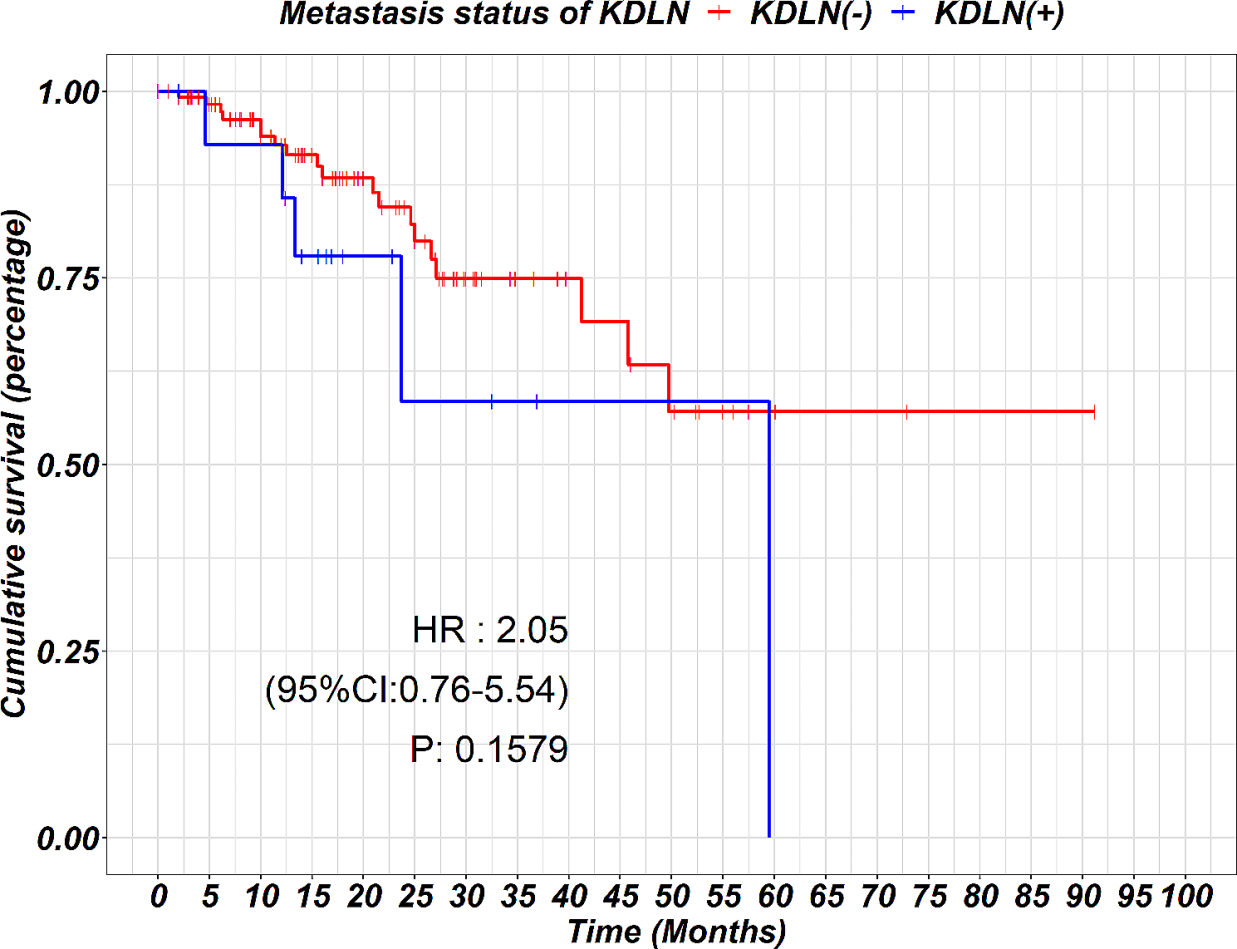
Kaplan–Meier curves displaying the overall survival of patients with key distal lymph node metastasis (blue line) compared to those without (red line)

In the survival analysis, there was no statistically significant difference between the subgroup with key distal lymph node metastasis (KDLN+) and the subgroup without (KDLN-) (3-year survival rate: 74.9% vs. 58.4%, *p* = 0.16), as depicted in Fig. 2.

## Discussion

In this study, we focused on the metastasis rate of the KDLN in gastric cancer patients after NAC. Our results indicate that even after NAC, the risk of key distal lymph node metastasis remains notably high, with a poor pathological response to NAC being a major risk factor. Proximal gastrectomy should not be recommended; instead, total gastrectomy with thorough distal lymphadenectomy is mandatory.

Lymph node metastasis is the most common form of metastasis in patients with locally advanced gastric cancer [18]. A thorough lymph node dissection is an important approach for complete tumor clearance [19]. For patients with lesions located in the upper part of the stomach, total gastrectomy with D2 lymph node dissection is commonly recommended, requiring thorough clearance of No. 1–7, 8a, 9, 11p, 11d, and 12a lymph nodes [2, 20, 21]. However, total gastrectomy significantly affects post-surgery quality of life (QoL), particularly regarding nutrition intake and physical function [22]. Proximal gastrectomy notably improves QoL compared to total gastrectomy. Nobuhiro et al. reported that proximal gastrectomy outperformed total gastrectomy in reducing body weight loss, the need for additional meals, and the occurrence of diarrhea and dumping syndrome. Consequently, proximal gastrectomy emerges as a valuable approach for preserving function in gastric cancer patients [4]. However, a major concern associated with this approach is the insufficient clearance of lymph nodes around the distal part of the stomach, specifically No. 4, 5, 6, and 12a lymph nodes (referred to as KDLN). Except for part of the No.4 lymph nodes, these lymph nodes are not routinely dissected during the proximal gastrectomy procedure. Therefore, if proximal gastrectomy were to be performed, it is critical to ensure no metastasis tumor cells are present in these lymph nodes. Previous study had found that in the early cases (cT1N0), the possibility of metastasis in KDLN is extremely low, therefore proximal gastrectomy could be a reasonable option [2, 23]. As for the cT2/3 cases, Masahiro Yura et al. found that even for cT2/3 cases, the chance of metastasis in the key distal lymph nodes is less than 1%, which is negligibly low [6]. Rui Peng, et al. also reported that when the tumor length is less than 4CM, the metastasis rate of KDLN is approximately zero in T2/3 cases, a propensity scores matching analysis also found no significant difference in overall survival between patients who received proximal gastrectomy or total gastrectomy, giving the conclusion that proximal gastrectomy can be indicated for patients with cT2/3 patients with tumor length less than 4CM [24]. For the cT4 cases, Sejin Lee, et al. found that serosal invasion, a macroscopic type IV tumor, and tumor size greater than 70 mm were risk factors indicating KDLN metastasis, thus proximal gastrectomy shall not be approached [25]. However, in the studies mentioned above, the conclusions were based on patients who received direct surgical resection, with no preoperative neoadjuvant chemotherapy. Nowadays, neoadjuvant chemotherapy has become an important approach for locally advanced gastric cancer, as recommended by several reputable guidelines. The National Comprehensive Cancer Network guideline recommend preoperative chemotherapy for cT2 or more and N any patients [21], the Chinese Society of Clinical Oncology recommend preoperative chemotherapy for locally advanced gastric cancer (cT2-4aN + M0) patients or (cT3-4N0M0) patients with tumor located in the esophago-gastric junction [26]. Since most gastric cancer patients are diagnosed at advanced stages in China [27], neoadjuvant chemotherapy is widely used. Neoadjuvant chemotherapy could potentially downstage or shrink the tumors, eliminate micro metastasis in the primary lesions as well as the lymph nodes [28]. In the FLOT trial [29], 15% of the patient achieve pathological complete response, meaning that no residual tumor in the primary site or lymph nodes, other relevant trials also reported a satisfactory response rate after NAC [30, 31], giving rise to the rationale that whether proximal gastrectomy could be indicated for selected patients with cT3/4 lesions after NAC. Therefore, our study aims to answer this question. We reviewed the grouped station lymph node details of 150 patients who received neoadjuvant chemotherapy and subsequent total gastrectomy from two high-volume tumor centers in China, all the patients were diagnosed with cT3/4 tumors located in the upper part of the stomach before treatment, after NAC, more than one fourth (28.7%) of the tumors were downstaged to T2 or below, 12.7% of the patients were even downstaged to T0. However, after investigating the metastatic status of the stational perigastric lymph nodes, we found a metastasis rate of 6%, 6.7%, and 2.7% in the No. 5, 6, and 12a lymph nodes, respectively. Additionally, we observed a therapeutic value index of 4 and 3.6 in the No. 5 and 6 lymph nodes, respectively. Though relatively lower compared to other grouped lymph nodes closer to the upper region of the stomach, these rates are significantly higher compared to the same grouped lymph nodes in the studies mentioned above, where the therapeutic value index of 5/6/12a was approximately zero. Moreover, when considered as one entity, the No. 5/6/12a (KDLN) has a metastasis rate of 10% and a therapeutic value index of 5.8, comparable to some of the proximal lymph nodes, asserting its value of dissection for survival.

There maybe a few explanations to it. Firstly, the cases in our study are mostly at advanced stages, more than half the patient (58.7%, 88/150) were with cT4 tumors before chemotherapy, serosal invasion is an important risk factor for KDLN metastasis, as proposed by Sejin Lee, et al [25]. Secondly, most of the cases are with large infiltrating tumor, 93% of the tumors were larger than 4CM, 27.3% invade the middle third of the stomach, both of which were risk factors associated with KDLN metastasis, as found by Ri et al [5]. Thirdly, unsatisfactory response to NAC may also be an important factor, as in the multivariate analysis, poor tumor regression grade emerged as an independent risk factor (OR 1.47, *p* = 0.04) associated with KDLN metastasis, poor TRG means insufficient clearance of the tumor, leading to higher probability of residual cell in the KDLN.

Another finding of our study is that the possibility of KDLN metastasis appears to be independent of the tumor length. In fact, the subgroup with a small tumor length (< 4CM) appears to exhibit a higher KDLN metastasis rate than the larger length group. However, considering there were only 11 cases in the smaller tumor subgroup (< 4CM), and another confounding such higher T stage (T4 64%,7/11), this result maybe biased. It can’t be concluded that patients with smaller tumors are prone to higher KDLN metastasis.

The overall survivals are not significantly different in the subgroups with or without KDLN metastasis, this maybe due to the fact that both subgroups received total gastrectomy, with thorough proximal and distal lymph node dissection, other treatment parameters such as the R0 resection rate, post-operative adjuvant chemotherapy were also comparable in the two subgroups, making the survival comparable.

To our knowledge, this is the first study investigating the oncological safety of proximal gastrectomy after neoadjuvant chemotherapy. Our results were based on data from two high cancer centers in China, which adds robustness and diversity to the conclusion. However, we acknowledge a few limitations to our study. Firstly, detailed information about the subgroups of the No.4 lymph node (i.e. No.4a/4sb/4d) is lacking, as No.4d is also an important distal lymph node in proximal gastrectomy. Arguably, incorporating information about the No.4d metastasis status would only result in an even higher KDLN metastasis rate, yet this would not change our final conclusion. Secondly, the cases in our study were all at advanced stages (cT3/4) before NAC due to the fact that NAC were applied to more advanced case in current practice, a detail investigation covering the cT2 group with smaller tumors is lacking. Thirdly, limited by the retrospective nature of the analysis and the sample size, we failed to identify the indications for proximal gastrectomy after NAC. KDLN metastasis only occurs in a small proportion of the patients, proximal gastrectomy may be a safe option for some patients under strictly limited indication. Although we found tumor response grade (TRG) and advanced ypTN associated with higher KDLN metastasis rates, these factors cannot be determined prior to surgery, which limit their use in patient selection. A well-designed prospective study is needed to accurately clarify the indications.

## Conclusions

For upper locally advanced gastric cancer patients with cT3/4 who underwent neoadjuvant chemotherapy, the risk of key distal lymph node metastasis remains notably high. Therefore, proximal gastrectomy should not be recommended; instead, total gastrectomy with thorough distal lymphadenectomy is mandatory for complete tumorclearance.

## Data Availability

The data that support the findings of this study are available from Dr. Junsheng Peng (E-mail: pengjsh@mail.sysu.edu.cn) upon reasonable request.

## Declarations

### Novelty and impact

The indication for proximal gastrectomy has expanded to include patients with proximal advanced gastric cancer. However, the indication for patients following chemotherapy remained unclear. This cohort study demonstrated that for proximal gastric cancer patients that underwent neoadjuvant chemotherapy, the metastasis rate of key distal lymph node can reach 10%, rendering proximal gastrectomy an unsafe choice.

### Tumor regression grade

Grade 0 (pathological complete response); Grade 1 (major response); Grade 2 (minor response); Grade 3 (no response or progression).

## Abbreviations

KDLN: Key distal lymph node
NAC: Neoadjuvant chemotherapy
CT: Computed tomography
TRG: Tumor regression grade
OR: Odd ratio
JGCA: Japanese Gastric Cancer Association
OS: Overall survival
LAGC: Locally advanced gastric cancer
QoL: Quality of life
