# Correction: Oncological risk of proximal gastrectomy for proximal advanced gastric cancer after neoadjuvant chemotherapy

**DOI:** 10.1186/s12885-024-12066-3

**Published:** 2024-03-06

**Authors:** Yonghe Chen, Xiaojiang Chen, Yi Lin, Shenyan Zhang, Zhiwei Zhou, Junsheng Peng

**Affiliations:** 1 Department of General Surgery, The Sixth Affiliated Hospital, Sun Yat-Sen University, 26 Yuancun Erheng Road, Guangzhou, 510655 China; 2 Guangdong Provincial Key Laboratory of Colorectal and Pelvic Floor Diseases, The Sixth Affiliated Hospital, Sun Yat-Sen University, Guangzhou, 510655 China; 3 Biomedical Innovation Center, The Sixth Affiliated Hospital, Sun Yat-Sen University, Guangzhou, 510655 China; 4 Department of Gastric Surgery, Sun Yat-Sen University Cancer Center, Guangzhou, 510060 China; 5 Department of Pathology, The Sixth Affiliated Hospital, Sun Yat-Sen University, Guangzhou, 510655 China

**Correction: BMC Cancer 24, 255 (2024)**

**https://doi.org/10.1186/s12885-024-11993-5**

Following publication of the original article [1], the authors reported an error in Fig. 1, specifically in colour spectrum within the heatmap located in the figure's lower right quadrant. The incorrect and the correct figures are supplied in this correction article.

**Incorrect Fig. 1** 
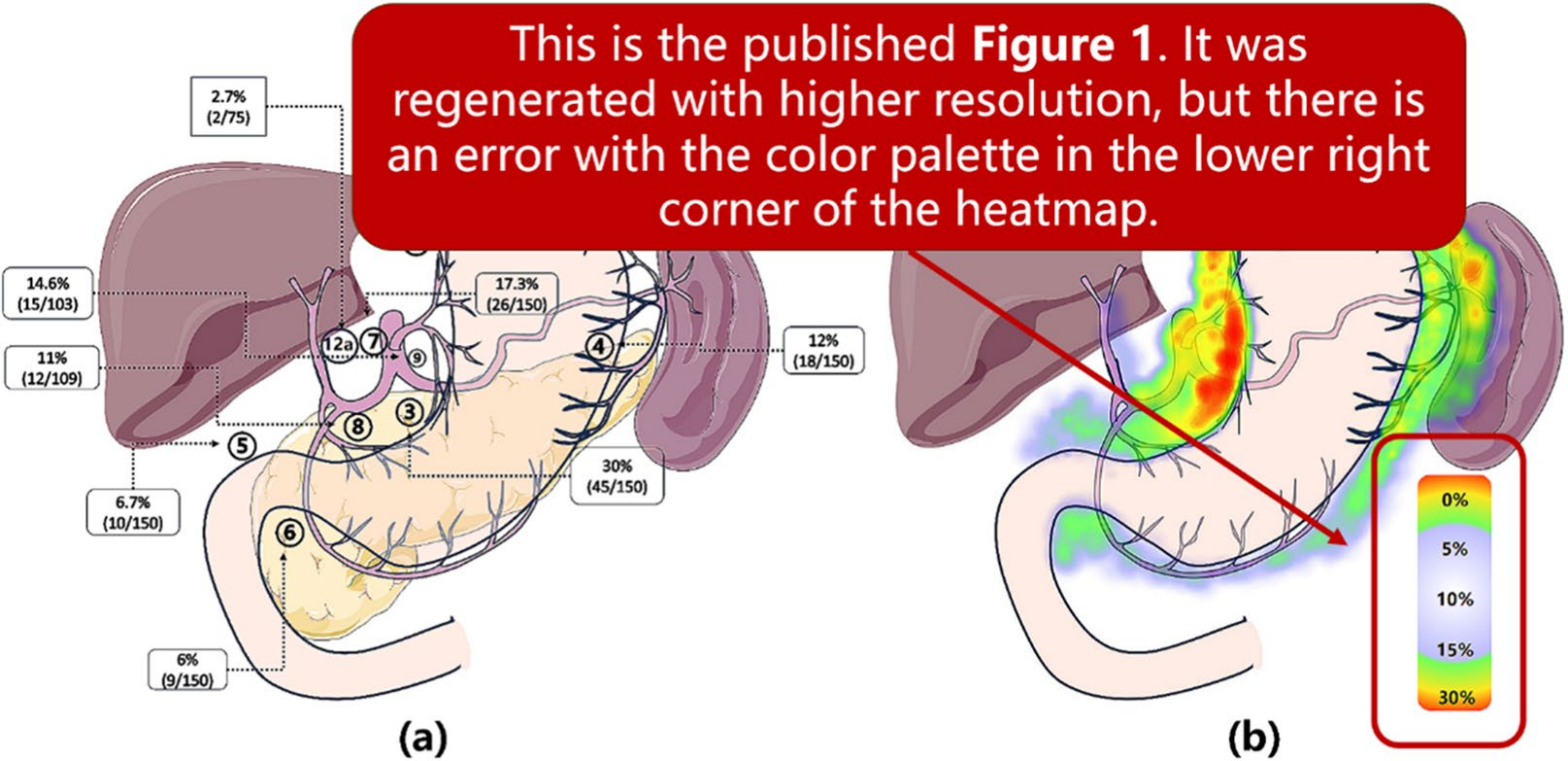


**Corrected Fig. 1**

**Fig. 1:**
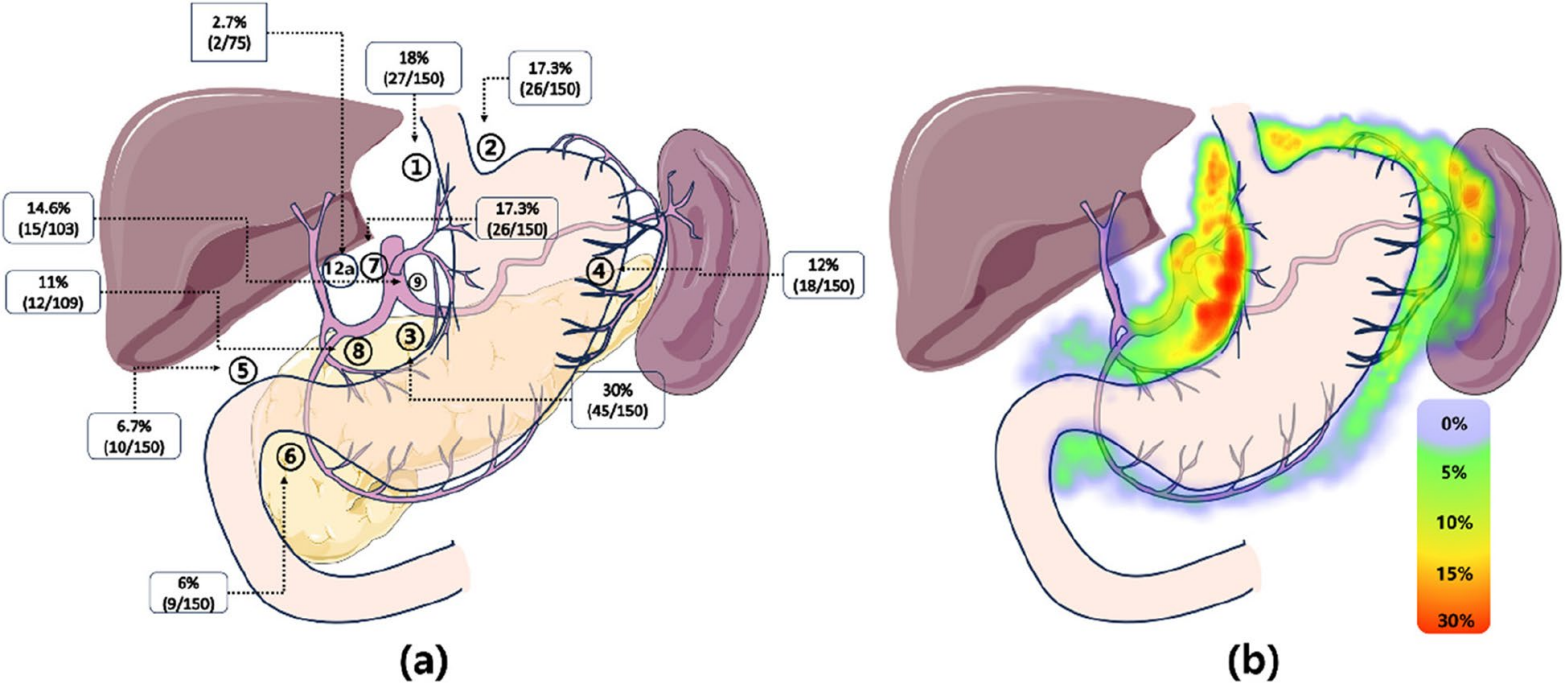
Q1 (**a**) This data map illustrates the metastasis rate of perigastric lymph nodes in proximal gastric cancer patients after neoadjuvant chemotherapy. Lymph nodes surrounding the proximal part of the stomach, such as No. 1/2/3/7, exhibit the highest metastasis rate (17.3% ~ 30%). Key distal lymph nodes, including No. 5/6/12a, have a collective metastasis rate of 10%. **b** This heatmap provides a visualization of the metastasis rate of grouped perigastric lymph nodes

The original article [1] has been corrected.

## References

[1] Chen Y, Chen X, Lin Y (2024). Oncological risk of proximal gastrectomy for proximal advanced gastric cancer after neoadjuvant chemotherapy. BMC Cancer.

